# From cantaloupe to cattle: Pseudomonas alabamensis sp. nov. described from diseased cantaloupe (Cucumis melo) foliage and a bovine (Bos taurus) nasopharynx

**DOI:** 10.1099/ijsem.0.006848

**Published:** 2025-07-14

**Authors:** Kiersten R. Fullem, Michelle P. MacLellan, Fanny B. Iriarte, Mousami Poudel, Sarah Capik, Keith Dedonder, James L. Bono, Dayna M. Harhay, Gregory P. Harhay, Erica M. Goss, Neha Potnis, Gerald V. Minsavage, Jeffrey B. Jones, Mathews L. Paret

**Affiliations:** 1 Department of Plant Pathology, University of Florida, Gainesville, FL, USA; 2 Department of Plant Pathology, University of Georgia, Tifton, GA, USA; 3 North Florida Research and Education Center, University of Florida, Quincy, FL, USA; 4 Tumbleweed Veterinary Services, PLLC, Amarillo, TX, USA; 5 Latham BioPharm Group, Elkridge, MD, USA; 6 USDA-ARS U.S. Meat Animal Research Center, Clay Center, NE, USA; 7 Department of Entomology and Plant Pathology, Auburn University, Auburn, AL, USA

**Keywords:** bacteria, cantaloupe, cattle, cucurbits, *Pseudomonas*

## Abstract

In 2022, a fluorescent bacterial isolate, designated 22-AL-CL-001, was isolated from diseased cantaloupe (*Cucumis melo*) foliage collected in Alabama, USA, displaying marginal necrosis and general blighting. Whole-genome sequencing and subsequent comparisons to publicly available bacterial genomes identified *Pseudomonas* isolate USDA-ARS-USMARC-56711, isolated from a clinically healthy calf (*Bos taurus*) in Kansas, USA, as genetically similar to isolate 22-AL-CL-001. The two isolates were then characterized using phenotypic and genetic analyses, including fluorescence on King’s medium B; LOPAT reactions; pathogenicity assays on cantaloupe, watermelon and squash plants; 16S rRNA gene sequence analysis; phylogeny based on multi-locus sequence analysis (MLSA) of four housekeeping genes; average nucleotide identity based on blast (ANIb); *in silico* DNA–DNA hybridization (isDDH) including comparison to the Type Genome Server and biochemical profiling using the Biolog Gen III microplate system. MLSA phylogeny placed both isolates into a single clade within the genus *Pseudomonas*, distant from all included reference strains. ANIb and isDDH analyses definitively identified the isolates as members of the same species (ANIb, 97.25; isDDH, 78.5%), though comparison scores to reference strains were all below the accepted thresholds for species determination. Biolog biochemical profiling, as well as MALDI-TOF mass spectrometry of isolate 22-AL-CL-001, was also unable to identify the isolate as a member of any existing bacterial species. Using a combination of genetic and phenotypic data, we conclude that the two isolates belong to a new species of *Pseudomonas*, for which we propose the name *Pseudomonas alabamensis*. The specific epithet, *alabamensis*, was chosen to represent the location where the type strain 22-AL-CL-001^T^ (NCPPB 4760^T^=LMG 33363^T^) was isolated, Alabama, USA. Taxonomic classification of the two isolates by the Genome Taxonomy Database revealed other previously sequenced bacterial strains that, according to ANI and isDDH results, also belong to *P. alabamensis*.

## Introduction

*Pseudomonas* is a large and ecologically significant bacterial genus, currently containing over 330 validly published species [1]. Members of the genus are genetically and phenotypically diverse and metabolically versatile, allowing them to inhabit an array of niches, including nearly all natural environments, as well as form intimate relationships with other living organisms, which may range from beneficial to parasitic [2,5]. Pseudomonads, in general, are greatly adaptable, and many are capable of switching between lifestyles, niches and hosts opportunistically. This is true of numerous plant-pathogenic *Pseudomonas* species, which can be found in a multitude of different environments, often spending part of their lifecycle as epiphytes or endophytes of plants, within seeds or in soils [2,5].

Remarkably, multiple species of plant-pathogenic or plant-associated *Pseudomonas* are also capable of inhabiting animal hosts. *Pseudomonas oryzihabitans* and *Pseudomonas aeruginosa*, which are primarily considered environmental saprophytes, are known to opportunistically infect humans as well as plants [6,9]. The two species cause nosocomial infections in humans, generally those who are immunocompromised or experiencing other illnesses, and *P. aeruginosa* is also associated with multiple diseases of other animals [3,6, 9,13]. Both are occasionally documented as plant pathogens, with *P. aeruginosa* causing general rot symptoms in such hosts as onion, sugar cane, lettuce and tobacco and *P. oryzihabitans* causing panicle blight of rice as well as stem and leaf rot and internal blackening of cantaloupe [8,9, 14,16]. *Pseudomonas fluorescens* and *Pseudomonas chlororaphis*, both members of the *P. fluorescens* species complex, are also known to be opportunistic human pathogens as well as saprophytic inhabitants of plant roots [17,18]. Additionally, epiphytic strains of the species *Pseudomonas syringae*, a prolific plant pathogen which infects many major crop species [19], have been documented as pathogenic, and sometimes lethal, to certain species of whiteflies and aphids [20].

While only a relatively small number of all described *Pseudomonas* species have been reported to infect or colonize both plants and animals, the versatility and opportunistic nature of the genus as a whole suggests that similar host associations within other species may exist undiscovered. As whole-genome sequencing of bacterial isolates becomes more prevalent and resulting sequence data are deposited in publicly available online databases, more associations between plant and animal-associated *Pseudomonas* isolates will likely be uncovered. In this paper, we present the novel species *Pseudomonas alabamensis* as an example of such an occurrence.

In June of 2022, a sample of diseased cantaloupe (*Cucumis melo*) foliage was collected from a commercial field experiencing marginal leaf necrosis and blighting in southern Alabama and brought to the University of Florida’s Plant Diagnostic Clinic in Quincy, Florida. Bacterial isolate 22-AL-CL-001, a fluorescent pseudomonad, was isolated from a necrotic lesion from the sample as part of a study identifying and characterizing pseudomonads associated with bacterial leaf spot of cucurbits (BLS) in the Southeastern United States [21]. While BLS is most commonly attributed to *P. syringae*, as well as a few additional plant-pathogenic *Pseudomonas* species [21], isolate 22-AL-CL-001 could not be identified as a member of any existing *Pseudomonas* species through phenotypic and genetic analyses. Comparison of 16S rRNA gene sequence data to the National Center for Biotechnology Information’s (NCBI) nucleotide database revealed a high level of genetic similarity within the region to that of *Pseudomonas* isolate USDA-ARS-USMARC-56711, which was, at that time, classified within the database as a member of the species *Pseudomonas monteilii*. Interestingly, the two isolates had come from very different isolation sources, with isolate USDA-ARS-USMARC-56711 having been collected in 2013 from a nasopharyngeal swab of a clinically healthy calf (*Bos taurus*) as part of an epidemiological study of bovine respiratory disease complex [22]. The calf in question had been sourced from a Tennessee sale barn, transported to Kansas overnight and sampled the next day, whereupon USDA-ARS-USMARC-56711 was recovered from the animal. As a result, the exact geographic origin of the isolate is uncertain, as it may have been acquired by the calf during transport from other calves, the trailer or another environmental source prior to transport. Comparisons of the two isolates using average nucleotide identity based on blast (ANIb) confirmed a high level of genetic similarity, as well as a relatively low percent ANI to the type strain of *P. monteilii*. And so, isolate USDA-ARS-USMARC-56711 was acquired by the University of Florida for characterization and further comparison to unidentified isolate 22-AL-CL-001.

In this paper, we describe the phenotypic and genetic methods used to characterize and identify the two isolates, including fluorescence, LOPAT testing [levan production, oxidase activity, pectolytic activity on potato, arginine dihydrolase activity and hypersensitive response (HR) on tomato and tobacco] [23], whole-genome sequencing, 16S rRNA gene sequence analysis, phylogeny based on multi-locus sequence analysis (MLSA) of four housekeeping genes, average nucleotide identity based on blast (ANIb), *in silico* DNA–DNA hybridization (isDDH), including comparison to the Type Genome Server (TYGS) and biochemical profiling with the Biolog Gen III microplate system. Additionally, we present further characterization of isolate 22-AL-CL-001 with transmission electron microscopy and matrix-assisted laser desorption/ionization time-of-flight mass spectrometry (MALDI-TOF MS) as well as comparisons of 22-AL-CL-001 and USDA-ARS-USMARC-56711 to genetically similar strains identified by the Genome Taxonomy Database (GTDB).

## Methods and results

### Phenotypic assays

Basic phenotypic assay results were consistently identical between isolates 22-AL-CL-001 and USDA-ARS-USMARC-56711. Both isolates were determined to be Gram-negative using a standard potassium hydroxide test [24] and produced round, bright yellow, glossy colonies when grown on nutrient agar (NA, Difco) for 48 h at 28 °C. When grown on King’s medium B for 24–48 h, the two isolates produced a diffusible pigment which fluoresced under UV light. LOPAT results for both isolates indicated negative reactions for levan production, oxidase activity, pectolytic activity on potato, arginine dihydrolase production and HR in both tomato and tobacco plants (Table 1).

**Table 1.** Fluorescence and LOPAT assay results for *P. alabamensis* strains as well as *P. monteilii*, the most closely related species as identified through ANIb analysis

	*P. alabamensis*		P. monteilii*
Phenotype	22-AL-CL-001^T^	USDA-ARS-USMARC-56711	DSM 14164^T^
Fluorescence	+	+	+
Levan	−	−	−
Oxidase	−	−	+
Pectolytic activity	−	−	nr
Arginine dihydrolase production	−	−	+
Hypersensitive response on tobacco	−	−	nr
Hypersensitive response on tomato	−	−	nr

Positive reactions are designated (+); negative reactions are designated (−); reactions which have not been reported are designated (nr).

* Data for *P. monteilii* acquired from Elomari *et al*. [39].

Isolates were also assessed for pathogenicity to cantaloupe (*Cucumis melo*), watermelon (*Citrullus lanatus*) and squash (*Cucurbita pepo* subsp. *pepo*) plants. Four-week-old seedlings were spray-inoculated until runoff with bacterial suspensions at a concentration of 10^8^ c.f.u. ml^−1^ and then placed into moistened polyethylene bags. Negative controls included completely untreated plants, plants mock-inoculated with sterile water and plants mock-inoculated with sterile water and placed into moistened polyethylene bags. *P. syringae* strain 13-139B, known to cause bacterial leaf spot on all three cucurbit hosts, was used as a positive control [25]. Each treatment consisted of a single pot, containing three seedlings, and was replicated in triplicate. Plants were allowed to incubate for 72 h under greenhouse conditions (day temperature, 28 °C; night temperature, 24 °C), after which, bags were removed, and plants were assessed for disease symptoms. Plants inoculated with the novel isolates exhibited no disease symptoms after 72 h. Positive control treatments exhibited typical BLS symptoms, while all negative control treatments were asymptomatic.

### Whole-genome sequencing and 16S rRNA gene sequence analysis

DNA extraction for isolate 22-AL-CL-001 was performed using the Wizard Genomic DNA Purification kit (Promega). DNA was sent to the Microbial Genome Sequencing Center (Pittsburgh, PA, USA) for whole-genome sequencing with the Illumina NextSeq 2000 platform. Resultant reads were assembled using a pipeline containing the genome assembly tool SPAdes (v. 3.10.1) [26]. Isolate USDA-ARS-USMARC-56711 had been previously sequenced using a PacBio RS II sequencer and P5 chemistry and then assembled using the PacBio Hierarchical Genome Assembly Process (HGAP3, v. 2.3.0). Genomes for both isolates were smaller than is typical of *Pseudomonas* species, with isolate 22-AL-CL-001 having a genome length of 4.66 Mb and isolate USDA-ARS-USMARC-56711 having a genome length of 4.70 Mb. However, checkM assays showed that both genomes had high completeness scores, indicating that the small genome sizes were not the result of sequencing error [27]. Genome statistics for both isolates are listed in Table 2. The 16S rRNA gene sequences of 22-AL-CL-001 and USDA-ARS-USMARC-56711 were compared using blast to NCBI’s nucleotide database, as well as a database containing 16S rRNA gene sequences from type strains of all validly published *Pseudomonas* species for which sequence data were available at the time of analysis (LPSN, *n*=329) [1]. The two isolates were found to share the highest level of 16S rRNA sequence similarity to each other, at 99.93% identity (Table 3). Both isolates shared the second-highest percent identity to *Pseudomonas parafulva* (strain DSM 17004^T^, 16S rRNA sequence accession number AB060132.1; 22-AL-CL-001, 99.72%; USDA-ARS-MARC-56711, 99.73%).

**Table 2.** Genome statistics, including genome coverage, genome length, N50 value and G+C content for *P. alabamensis* isolates. Completeness and contamination scores were calculated using CheckM (v. 1.1.2, default parameters) [27]

Isolate	22-AL-CL-001^T^	USDA-ARS-USMARC-56711
Genome coverage	91	113
Genome length (Mb)	4.66	4.7
N50 value	267674	4714359
G+C content (mol%)	64.5	64.5
Number of contigs	67	1
Completeness (%)	99.58	99.57
Contamination	0.48	0.62

**Table 3.** Genomic relationships between *P. alabamensis* strains and type strains of the most closely related *Pseudomonas* species as identified by genetic comparisons and other *Pseudomonas* species known to inhabit both plant and animal hosts

		16S rRNA gene sequence similarity **(%)**		ANIb **(%)**		isDDH **(%)**	
Strains	NCBI accession	1*	2	1*	2	1*	2
***P. alabamensis* 22-AL-CL-001^T^**	GCF_030580815.1	100	99.93	100	97.25	100	78.5
***P. alabamensis* USDA-ARS-USMARC-56711**	GCA_001534745.1	99.93	100	97.25	100	78.5	100
**Most closely related species**							
*P. monteilii* DSM 14164^T^	GCF_000621245.1	98.2	98.2	81.2	81.21	24.8	24.8
*P. peradeniyensis* BW13M1^T^	GCF_014268935.2	98.68	98.71	80.68	80.74	25.9	26.3
*P. muyukensis* COW39^T^	GCF_019139535.1	98.96	99.02	80.29	80.33	26	26
*P. xantholysinigenes* RW9S1A^T^	GCF_014268885.2	98.82	98.89	80.23	80.3	25.9	26
*P. sichuanensis* WCHPs060039^T^	GCF_003231305.1	98.68	98.77	80.09	80.07	26.2	26.3
*P. carassii* 137^T^	GCF_036324045.1	98.68	98.71	80.07	80.07	25.9	26.1
*P. plecoglossicida* NBRC 103162^T^	GCF_030160515.1	98.89	98.93	80.06	80.1	25	25.9
*P. oryziphila* 1257^T^	GCF_003940825.1	98.84	98.84	79.98	80	26.2	26.4
*P. asiatica* RYU5^T^	GCF_009932335.1	99.03	99.09	79.98	79.9	26	26.2
*P. fakonensis* COW40^T^	GCF_019139895.1	98.82	98.89	79.86	79.88	25.9	25.9
*P. maumuensis* COW77^T^	GCF_019139675.1	98.96	99.02	79.82	79.8	25.4	25.7
*P. wayambapalatensis* RW3S1^T^	GCA_014268975.2	98.91	98.97	79.62	79.67	25.2	25.4
*P. soli* LMG 27941^T^	GCF_900110655.1	98.84	98.89	79.54	79.56	25.1	25.3
*P. juntendi* BML3^T^	GCF_009932375.1	99.1	99.15	79.4	79.45	24.9	25.2
*P. boreofloridensis* K13^T^	GCF_030580795.1	99.65	99.55	79.39	79.33	24.7	24.8
*P. reidholzensis* CCOS 865^T^	GCF_900536025.1	98.54	98.54	79.38	79.33	25.5	25.6
*P. taiwanensis* DSM 21245^T^	GCF_000425785.1	98.89	98.91	79.36	79.29	25.8	25.1
*P. vlassakiae* RW4S2^T^	GCF_014269035.2	99.02	99.22	79.33	79.32	25.3	25.3
*P. inefficax* JV551A3^T^	GCF_900277125.1	98.96	99.02	79.32	79.4	26	26.3
*P. kurunegalensis* RW1P2^T^	GCF_014269245.2	99.05	99.16	79.28	79.17	24.9	25.1
*P. urmiensis* SWRI10^T^	GCF_014268815.2	99.03	99.09	79.08	79.12	25.2	25.3
*P. parafulva* DSM 17004^T^	GCF_000425765.1	99.72	99.73	78.85	78.83	24.2	24.4
*P. promysalinigenes* RW10S1^T^	GCF_014269025.2	99.02	99.16	78.66	78.75	24.3	24.5
*P. palmensis* BBB001^T^	GCF_017848315.1	97.78	97.92	78.47	78.39	24.1	24.2
*P. fulva* DSM 17717^T^	GCF_000621265.1	98.96	98.95	78.45	78.49	23.9	24.1
*P. laurentiana* JCM 32154^T^	GCF_014648275.1	99.09	99.12	77.12	77.13	22.7	22.8
*P. chlororaphis* ATCC 9446^T^	GCF_028747405.1	97.1	97.1	76.56	76.6	22.7	22.8
**Species known to inhabit both plants and animals**							
*P. fluorescens* NCTC 10038^T^	GCF_900475215.1	96.61	96.84	75.19	75.28	21.8	22
*P. aeruginosa* DSM 50071^T^	GCF_012987025.1	95.57	95.46	75.05	75.03	21.7	21.5
*P. syringae* ICMP 3023^T^	GCF_000507185.2	96.82	96.97	74.57	74.64	21.3	21.7
*P. oryzihabitans* DSM 6835^T^	GCF_012986195.1	98.47	98.56	74.02	73.66	21	21

*1, *P. alabamensis* 22-AL-CL-001T; 2, *P. alabamensis* USDA-ARS-USMARC-56711.

### Phylogenetic analysis based on multi-locus sequence data

**Fig. 1.**
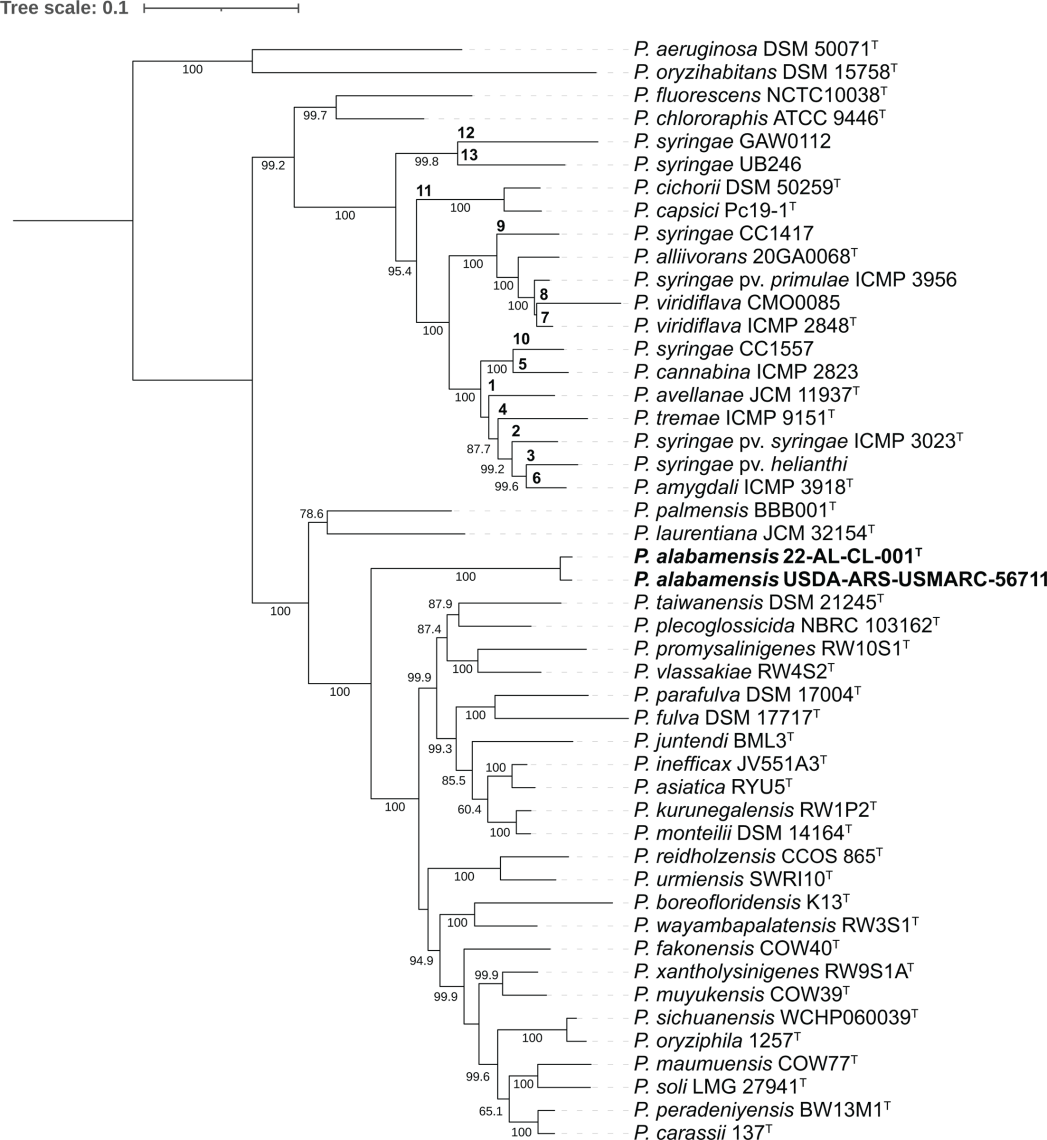
Maximum likelihood phylogeny inference of *P. alabamensis* and reference strains, including the most closely related species as identified by genetic comparisons, as well as representatives of *P. syringae* phylogroups and species known to inhabit both plant and animal hosts, based on concatenated alignments of the housekeeping genes *gltA*, *rpoD*, *gapA* and *gyrB*. Bootstrap values based on 100 replicates are indicated at branching points. Bolded numbers designate Pssc phylogroups.

The *P. syringae* species complex (Pssc) is a large phylogenetic group that contains many plant-pathogenic and plant-associated *Pseudomonas* species, including *P. syringae*, the most common causal agent of BLS, and several additional *Pseudomonas* species associated with leaf spot diseases of cucurbit crops [21,28]. This complex has been divided into 13 phylogroups based on MLSA using the four housekeeping genes *cts* (*gltA*), *rpoD*, *gapA* and *gyrB* [28,29]. To determine the phylogenetic placement of the two isolates from this study within the *Pseudomonas* genus, and in relation to the Pssc, MLSA based on these four gene sequences was performed using the program AutoMLSA2 (v. 0.8.1) [30]. The two isolates were compared to *Pseudomonas* strains representing the 13 phylogroups of the Pssc, species to which they shared relatively high 16S rRNA sequence similarity, as well as other *Pseudomonas* species also known to infect both plant and animal hosts. Analysis was performed using reference genomes obtained from NCBI and gene sequences from the Plant Associated and Environmental Microbes Database (pamdb.org). A maximum likelihood phylogenetic tree was constructed using IQ-TREE (v. 2.1.3) [31,32] and visualized using ITOL (v. 6.9) [33] and Adobe Illustrator (Fig. 1). In this phylogeny, isolates 22-AL-CL-001 and USDA-ARS-USMARC-56711 were placed into a single clade, which was located outside of the Pssc and was distant from all other *Pseudomonas* reference strains included in the analysis. These results show that the novel isolates are phylogenetically distant from both the Pssc, which contains *Pseudomonas* species typically associated with leafspot diseases of cucurbits, as well as other *Pseudomonas* species known to infect both plant and animal hosts.

### Whole-genome comparisons

ANIb and isDDH analyses were conducted using whole genomes of isolates 22-AL-CL-001 and USDA-ARS-USMARC-56711 as well as type strains of *Pseudomonas* species to which they had high 16S rRNA sequence similarity or relative phylogenetic proximity. The established threshold for species determination is considered to be greater than or equal to 95% for ANI analysis and greater than or equal to 70% for DDH [34]. ANIb analysis was performed using the Python module pyani (v. 0.2.12) [35], while isDDH was conducted using the web tool Genome-Genome Distance Calculator (v. 3.0, formula 2, http://ggdc.dsmz.de/home.php) [36,37]. For both analyses, the two isolates were found to have the highest percent similarity to each other with an ANIb score of 97.25% and an isDDH score of 78.5%, identifying them as members of the same species (Table 3). Neither isolate was found to share greater than 95% ANIb or greater than 70% isDDH to any reference strain tested, suggesting that they belong to a currently undescribed species of *Pseudomonas*. Genetically, the most closely related *Pseudomonas* species to the two isolates, as determined by ANIb and isDDH, were still relatively distant. *P. monteilii* (type strain DSM 14164^T^) was the top ANIb match to both 22-AL-Cl-001 and USDA-ARS-USMARC-56711, though comparisons to the isolates produced scores of only 81.2% and 81.21%, respectively. For isDDH, *P. oryziphila* (type strain 1257^T^) was identified as the closest species match to the isolates, producing similarly low scores of 26.2% (22-AL-CL-001) and 26.4% (USDA-ARS-USMARC-56711).

### Comparison to the TYGS

To further verify that the isolates did not belong to any currently described prokaryotic species, the genomes of 22-AL-CL-001 and USDA-ARS-USMARC-56711 were compared to the online Type Genome Server (https://tygs.dsmz.de/) [38]. The TYGS is a high-throughput web tool that compares user-uploaded genomes to a database of prokaryotic type strain genomes using isDDH for species identification. The TYGS was unable to identify either isolate as a member of any species contained within its database, supporting their identities as members of a new species. The results of whole-genome and 16S rRNA gene sequence-based phylogenies produced by the TYGS were overall consistent with those of the MLSA phylogeny. These phylogenetic trees are included within the supplementary materials (Figs S1 and S2, available in the online Supplementary Material).

Based on the results of the genetic and phylogenetic analyses described above, we propose the placement of isolates 22-AL-CL-001^T^ and USDA-ARS-USMARC-56711 within a novel species of *Pseudomonas*, named *P. alabamensis* after the geographical origin of isolate 22-AL-CL-001^T^ (Alabama, USA).

### Physiological and chemotaxonomic analyses

Isolates 22-AL-CL-001^T^ and USDA-ARS-USMARC-56711 were further characterized with biochemical profiling using the Biolog Gen III microplate system. Overnight cultures, grown at 28 °C on Biolog universal growth agar (BUG, Biolog Inc.), were used to create bacterial suspensions of the recommended level of turbidity in Biolog inoculation fluid. For each isolate, a microplate was then inoculated with 100 µl of suspension per well and incubated at 28 °C for 24 h, after which, test results were recorded and compared to the Biolog database (Table S2). Biolog software identified both isolates as members of the genus *Pseudomonas*, though it was unable to further identify either as a member of any existing *Pseudomonas* species contained within its database (MicroLog M System, v. 5.1.1). Of the species contained within the Biolog database, *P. fluorescens* (22-AL-Cl-001^T^) and *Pseudomonas plecoglossicida* (USDA-ARS-USMARC-56711) were identified as the closest matches to the novel isolates. Comparison of the biochemical profile of isolate 22-AL-CL-001^T^ to that of *P. fluorescens* produced a similarity score of 0.157, while comparison of isolate USDA-ARS-USMARC-56711’s profile to that of *P. plecoglossicida* produced a similarity score of 0.206, with both scores being significantly lower than the accepted value of ≥0.5 necessary for species determination. Carbon utilization data for * P. monteilii*, the most closely related species according to ANIb analysis, were also obtained and compared to Biolog profiles of * P. alabamensis* strains, revealing differences in utilization patterns between the two species (Table S2) [39].

Isolate 22-AL-CL-001^T^, which was designated as the type strain of *P. alabamensis* sp. nov., was further analysed using MALDI-TOF MS performed by Charles River Laboratories (Newark, DE, USA). Resulting spectra were compared to Bruker Biotyper (v. 11758) and Charles River (v. 23.01) spectral reference libraries for species identification. Charles River Laboratories bases probable species identification on a similarity score between a tested isolate and a reference strain of greater than or equal to 1.75. Isolate 22-AL-CL-001^T^ did not produce a similarity score within this range to any examined reference strain and, so, was unable to be identified as any existing bacterial species based on MALDI-TOF MS.

**Fig. 2.**
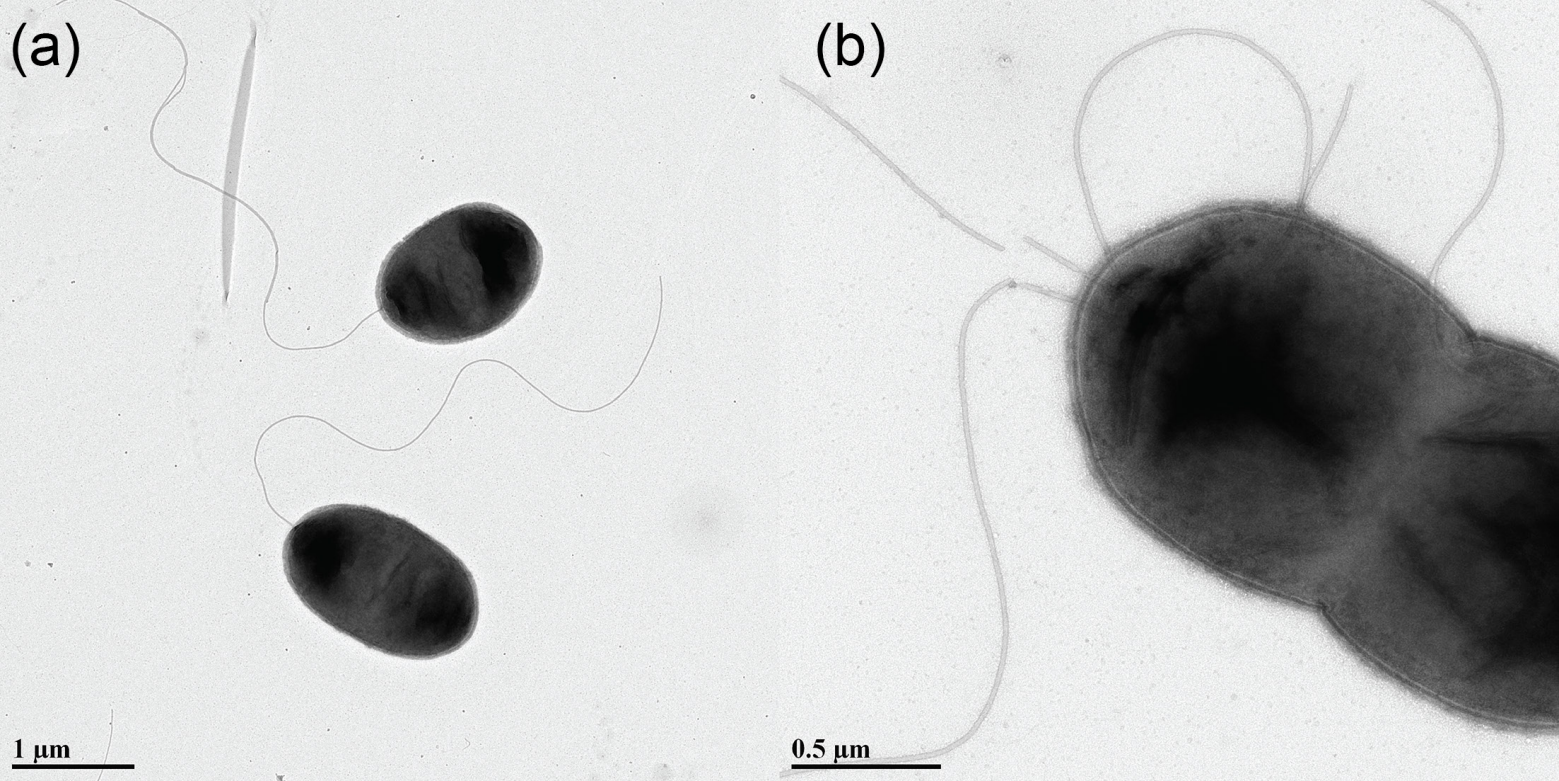
Images of (a) monoflagellate and (b) multiflagellate cells of *P. alabamensis* 22-AL-CL-001^T^ as captured by a Tecnai G2 Spirit TWIN 120 kV transmission electron microscope, University of Florida’s ICBR.

Bacterial cells of isolate 22-AL-CL-001^T^ were imaged using transmission electron microscopy, conducted by the University of Florida’s Interdisciplinary Center for Biotechnology Research (ICBR), using a Tecnai G2 Spirit TWIN 120 kV microscope. Cells were observed to be rod-shaped, slightly rounded, and to possess one or more polar flagella (Fig. 2).

### Comparison to the genome taxonomy database

To identify whether any additional strains of *P. alabamensis* sp. nov had been previously sequenced and made publicly available online, the classification of isolates 22-AL-CL-001^T^ and USDA-ARS-USMARC-56711 within the Genome Taxonomy Database was determined [40]. The GTDB is a database that imports prokaryotic genome data from the NCBI Assembly database and organizes them into ‘species clusters’ on the basis of average nucleotide identity, allowing for the identification of genetically similar isolates. Perusal of the database revealed that both *P. alabamensis* isolates identified in this study had been placed within the GTDB species cluster *Pseudomonas_E monteilii_A*, alongside seven additional genomes, including six classified within NCBI as *P. monteilii* and one as *P. entomophila*. The type strains of these two species (*P. monteilii*: NBRC 103158^T^=DSM 14164^T^; *P. entomophila*: L48^T^) did not appear within the *Pseudomonas_E monteilii*_A cluster and were instead sorted by GTDB into two separate species clusters, *Pseudomonas*_E *monteilii* and *Pseudomonas*_E *entomophila*, respectively. USDA-ARS-USMARC-56711 had been declared by GTDB as the representative strain of species cluster *Pseudomonas_E monteilii*_A. Results of ANI, isDDH and MLSA comparisons of the isolates belonging to *Pseudomonas*_E *monteilii*_A, as well as type strains of *P. monteilii* and *P. entomophila*, are included within the supplementary materials (Table S1, Fig. S3). Both ANI and isDDH analyses revealed that the seven additional isolates belong to *P. alabamensis*, with an average ANI to type strain 22-AL-CL-001^T^ of 97.57% and an average isDDH score of 78.85%. None of these seven isolates shared ANI or isDDH values above the established thresholds for species identification to the type strains of *P. monteilii* or *P. entomophila*. Further inspection of the NCBI assembly records of the seven additional isolates revealed that taxonomy checks performed by NCBI had produced inconclusive results, supporting the conclusion that these organisms had been previously described as members of *P. entomophila* or *P. monteilii* in error. Additionally, all seven isolates were found to have relatively short genomes (average=4.60 Mb), similar in size to those of 22-AL-CL-001^T^ and USDA-ARS-USMARC-56711.

MLSA was performed as previously described and included strains contained within GTDB species cluster *Pseudomonas*_E *monteilii_A*, type strain genomes of *P. monteilii* and *P. entomophila*, and the ten *Pseudomonas* species determined to have the highest percent ANI to *P. alabamensis* type strain 22-AL-CL-001^T^, with *P. syringae* type strain ICMP 3023^T^ included as an outgroup. The results of the MLSA phylogeny were consistent with those of the ANI and isDDH analyses and showed that isolates 22-AL-CL-001^T^ and USDA-ARS-USMARC-56711 clustered with the seven additional isolates placed within the *Pseudomonas_E monteilii*_A species group by the GTDB and that this group was phylogenetically distinct from all other *Pseudomonas* species used in the analysis, including the type strains of *P. monteilii* and *P. entomophila*.

NCBI metadata associated with the genomes of the additional seven GTDB isolates show that they had been collected within a similar time frame to isolates 22-AL-CL-001^T^ and USDA-ARS-USMARC-56711, between 2017 and 2022, though from different sources, including from metal and other unspecified environmental sources. All seven of the additional isolates had also been collected from the USA, specifically, from the states of New York and North Carolina (Table S1).

## Conclusion

In conclusion, we propose that isolates 22-AL-CL-001^T^ and USDA-ARS-USMARC-56711 represent a novel bacterial species, designated *P. alabamensis*, based on 16S rRNA gene sequence, ANIb and isDDH analyses, including comparison to the TYGS; phylogenies based on MLSA, 16S rRNA gene sequence analysis and whole-genome comparisons; biochemical profiling with the Biolog Gen III microplate system; and MALDI-TOF MS analysis. This species is notable as an example of a pseudomonad that has been isolated from both plant and animal hosts.

## Description of *Pseudomonas alabamensis* sp. nov.

*Pseudomonas alabamensis* (al.a.bam.en’sis. N.L. fem. adj. *alabamensis*, pertaining to the geographical location of isolation of type strain 22-AL-CL-001^T^, Alabama, USA).

Cells are Gram-negative motile rods (1.9–2.3 µm in length and 1.1–1.5 µm in width) with one or more polar flagella. Cells are slightly rounder than is typical of *Pseudomonas* species. Colonies, when grown on NA at 28 °C for 48 h, are bright yellow in color, glossy, round and ~3.0 mm in diameter. Isolates produce a diffusible fluorescent pigment when grown on King’s medium B; are negative for levan production, oxidase activity, pectolytic activity on potato and arginine dihydrolase activity; and do not elicit an HR in either tobacco or tomato plants. Isolates were not observed to cause disease when spray-inoculated on cantaloupe (*Cucumis melo*), watermelon (*Citrullus lanatus*) and squash (*Cucurbita pepo* subsp. *pepo*) seedlings. Isolates were observed to grow at pH levels of 5 and 6 as well as at salinity levels of 1%, 4% and 8% NaCl. Growth of isolate 22-Al-Cl-001^T^ suspended in tryptic soy broth (Sigma-Aldrich) was assessed at incubation temperatures of 0, 4, 15, 21, 28, 32, 37 and 41 °C. After 48 h of incubation, growth was observed only in cultures incubated at temperatures between 15 and 37 °C, with optimal growth occurring between 21 and 32 °C. Type strain 22-AL-CL-001^T^ (NCPPB 4760=LMG 33363) was isolated from cantaloupe foliage displaying marginal leaf necrosis and blighting in Alabama, USA. Isolate 22-AL-CL-001^T^ has a genome size of 4.66 Mb and a G+C content of 64.5 mol%. NCBI accession numbers for *P. alabamensis* genomes are as follows: 22-AL-Cl-001^T^, GCA_030580815.1; USDA-ARS-USMARC-56711, GCA_001534745.1. Accession numbers for 16S rRNA gene sequences are: 22-AL-Cl-001^T^, OR725053.1; USDA-ARS-USMARC-56711, PQ115154.1.

## Supplementary material

10.1099/ijsem.0.006848 Uncited Fig. S1.

## Abbreviations

ANIb: average nucleotide identity based on blast
GTDB: Genome Taxonomy Database
HR: hypersensitive response
ICBR: Interdisciplinary Center for Biotechnology Research
isDDH: *in silico* DNA–DNA hybridization
LOPAT: levan production, oxidase activity, pectolytic activity on potato, arginine dihydrolase activity and hypersensitive response on tomato and tobacco
MALDI-TOF: matrix-assisted laser desorption/ionization time of flight
MLSA: multi-locus sequence analysis
NCBI: National Center for Biotechnology Information
Pssc: *Pseudomonas syringae* species complex
TYGS: Type Genome Server
