# Supplementary material for: From cantaloupe to cattle: Pseudomonas alabamensis sp. nov. described from diseased cantaloupe (Cucumis melo) foliage and a bovine (Bos taurus) nasopharynx
Source: Int J Syst Evol Microbiol. 2025 Jul 14;75(7):006848. doi: 10.1099/ijsem.0.006848 (PMC12281984; doi:10.1099/ijsem.0.006848)
Supplement: Uncited Fig. S1. [file ijsem-75-06848-s001.pdf]

Supplementary Figure 1. A 16S rRNA gene sequence phylogeny including strains of *P. alabamensis* and relevant *Pseudomonas* type strain genomes as produced by the online Type Genome Server (TYGS, https://tygs.dsmz.de/)

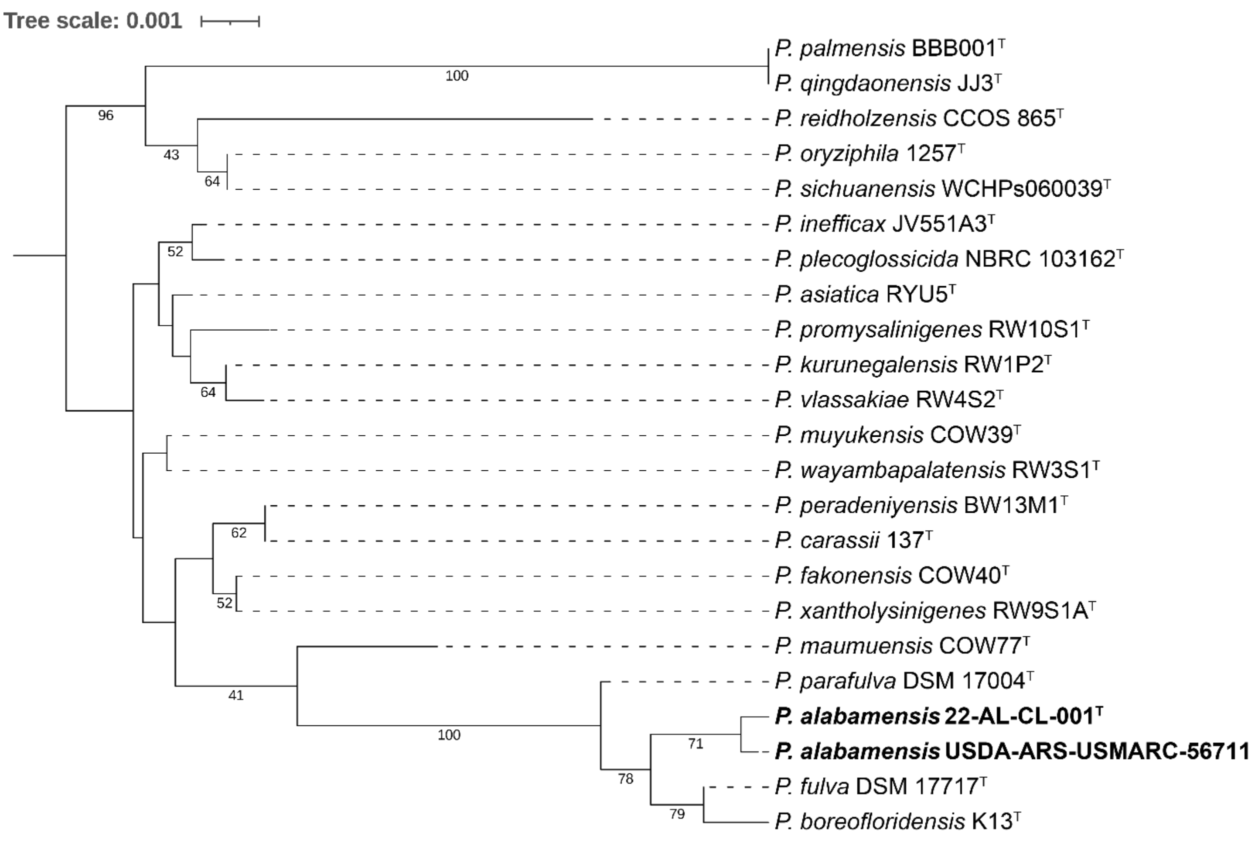

Phylogeny generated using distance formula 5 and distance algorithm CharacterCoverage.

Bootstrap values based on 100 replicates are indicated at branch points.

Supplementary Figure 2. A whole-genome phylogeny based on isDDH analysis including strains of *P. alabamensis* and relevant *Pseudomonas* type strain genomes as produced by the online Type Genome Server (TYGS, https://tygs.dsmz.de/)

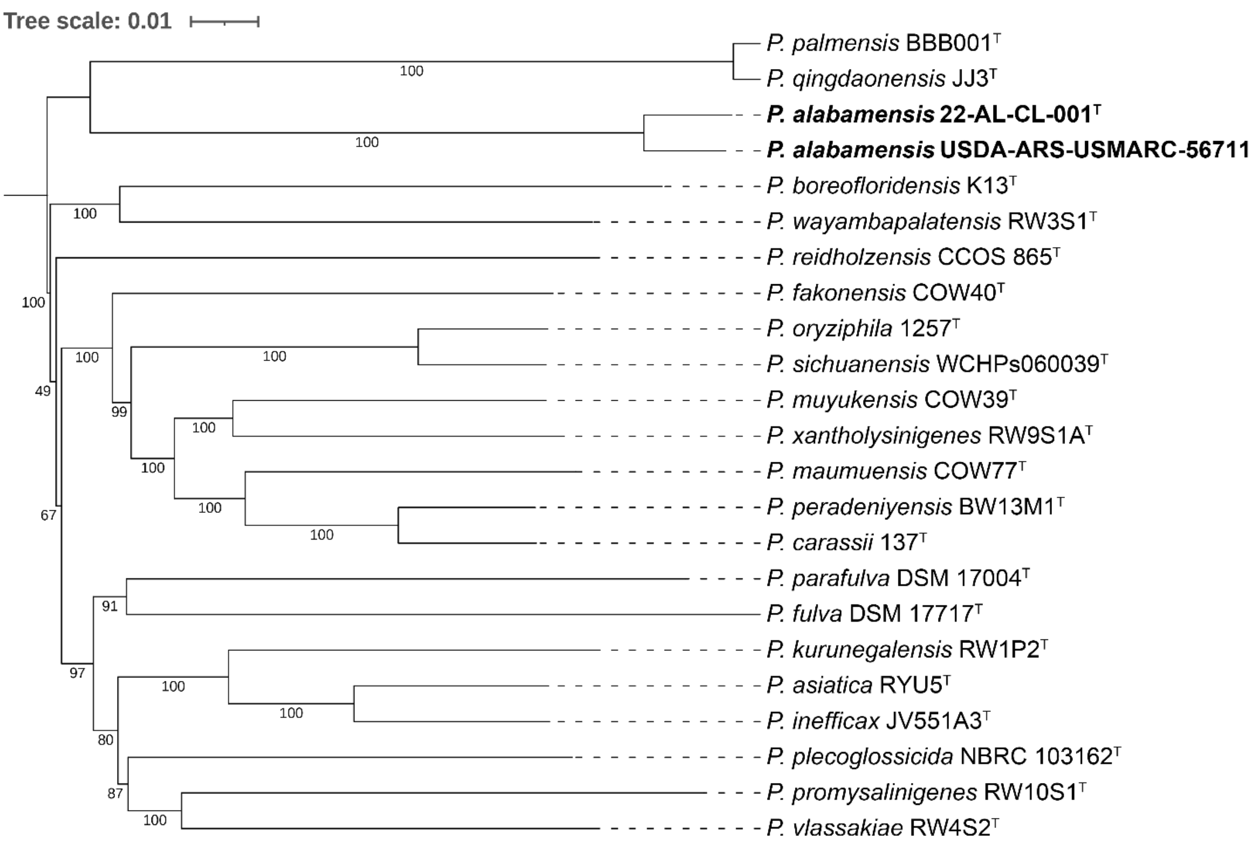

Phylogeny generated using distance formula 5 and distance algorithm GreedyWithTrimming.

Bootstrap values based on 100 replicates are indicated at branch points.

Supplementary Table 1. Metadata and genetic comparisons of isolates identified by the Genome Taxonomy Database (GTDB) as belonging to species cluster *Pseudomonas*_E *monteilii*_A

Isolate	NCBI Taxonomy*	GTDB Taxonomy	NCBI Accession	Submitter	Year of Isolation	Location of Isolation	Isolation Source	Genome Size (Mb)	Genome Completeness (CheckM)	ANIb (%)				isDDH (%)			
										1**	2	3	4	1**	2	3	4
***P. alabamensis* 22-AL-CL-001^{T}**	*P.* sp. 22-AL-CL-001	*Pseudomonas_E monteilii_A*	GCF_030580815.1	University of Florida	2022	AL, USA	Cantaloupe Plant	4.66	99.58	100	97.25	81.2	82	100	78.5	24.8	25.7
***P. alabamensis* USDA-ARS-USMARC-56711**	*P.* sp. 22-AL-CL-001	*Pseudomonas_E monteilii_A*	GCA_001534745.1	USDA-ARS-USMARC	2016	KS, USA	Bovine Mucus	4.7	99.57	97.25	100	81.21	82.17	78.5	100	24.8	25.9
P. monteilii UBA3487	*P. monteilii*	*Pseudomonas_E monteilii_A*	GCA_002377825.1	University of Queensland	2017	New York City, NY, USA	Metal	3.73	77.66	97.25	97.23	81.08	81.80	77.3	76.8	24.6	25.6
P. monteilii DE0603	*P. monteilii*	*Pseudomonas_E monteilii_A*	GCA_007665385.1	BGI	2019	Durham, NC, USA	Environmental	4.78	99.58	97.64	97.96	81.27	82.07	79.3	82.2	24.8	25.7
P. monteilii DE0601	*P. monteilii*	*Pseudomonas_E monteilii_A*	GCA_007665425.1	BGI	2019	Durham, NC, USA	Environmental	4.76	99.58	97.63	97.97	81.27	82.11	79.2	82.2	24.8	25.7
P. monteilii DE0600	*P. monteilii*	*Pseudomonas_E monteilii_A*	GCA_007665435.1	BGI	2019	Durham, NC, USA	Environmental	4.75	99.58	97.64	97.99	81.22	82.03	79.2	82.2	24.8	25.7
P. monteilii DE0596	*P. monteilii*	*Pseudomonas_E monteilii_A*	GCA_007665485.1	BGI	2019	Durham, NC, USA	Environmental	4.75	99.58	97.64	97.99	81.32	82.10	79.2	82.2	24.8	25.7
P. monteilii DE0591	*P. monteilii*	*Pseudomonas_E monteilii_A*	GCA_007665585.1	BGI	2019	Durham, NC, USA	Environmental	4.75	99.58	97.63	97.96	81.28	82.08	79.2	82.2	24.8	25.7
P. entomophila RIT-PI-AB	*P. entomophila*	*Pseudomonas_E monteilii_A*	GCF_025642795.1	Rochester Institute of Technology	2022	Wheatland, NY, USA	N/A	4.7	99.57	97.54	98.44	81.24	82.02	78.6	86.6	24.8	25.7

*NCBI taxonomy check results for all isolates, excepting *P. alabamensis* isolates 22-AL-CL-001^{T} and USDA-ARS-USMARC-56711, were listed as "inconclusive" within the NCBI genome database, contradicting their current classification as members of *P. entomophila* or *P. monteilii*.

**1: *P. alabamensis* 22-AL-CL-01^{T}; 2: *P. alabamensis* USDA-ARS-USMARC-56711; 3. *P. monteilii* 14164^{T}; 4: *P. entomophila* L48^{T}

Supplementary Figure 3. Maximum likelihood phylogeny inference of isolates placed within the *Pseudomonas*_E *monteilii*_A species cluster by the Genome Taxonomy Database (GTDB) and reference strains, based on concatenated alignments of the housekeeping genes *gltA*, *rpoD*, *gapA*, and *gyrB*. Bootstrap values based on 100 replicates are indicated at branching points.

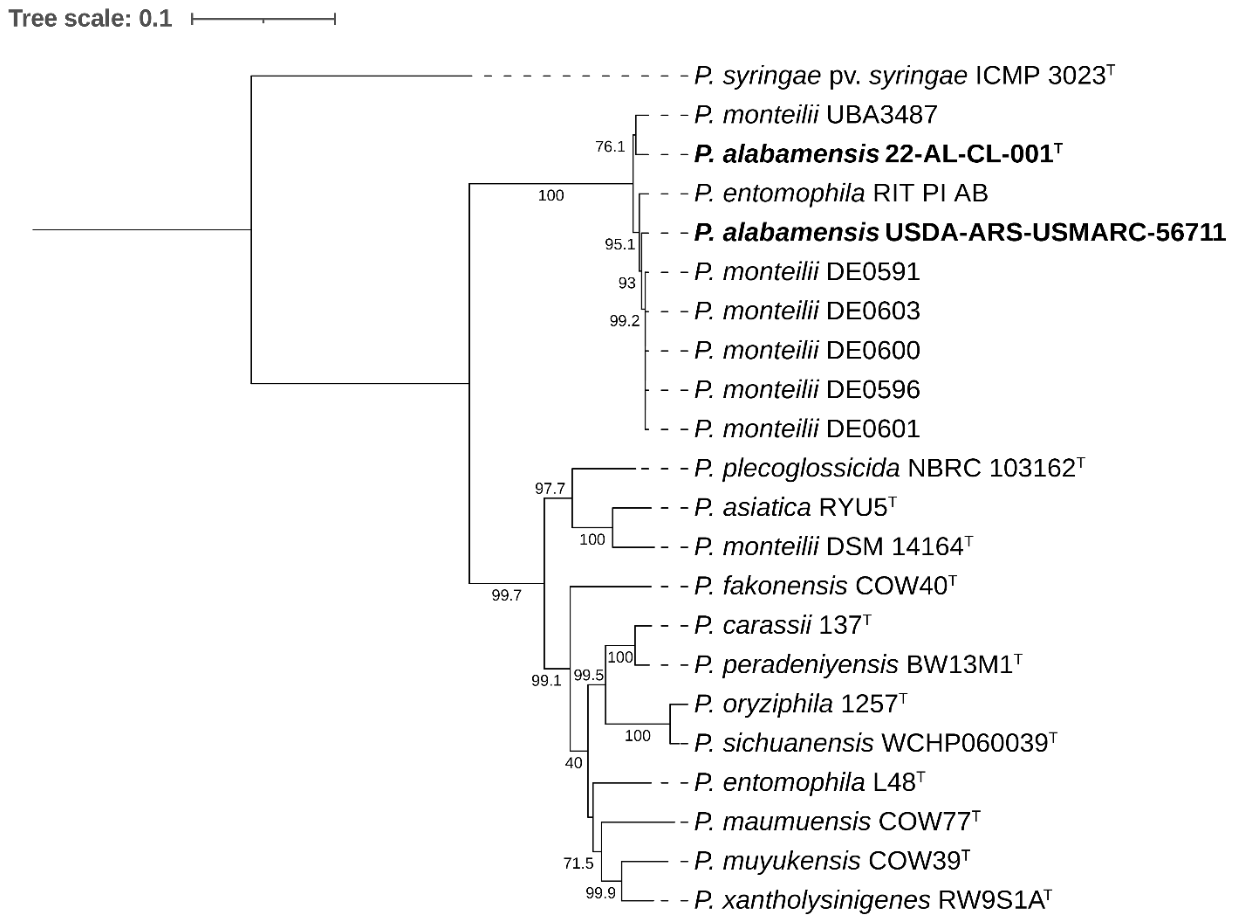

Supplementary Table 2. Biolog Gen III biochemical profiling results for *P. alabamensis* isolates 22-AL-CL-001ᵀ and USDA-ARS-USMARC-56711, as well as the Biolog profiles of the closest species matches as identified by Biolog software (MicroLog™ M System, v. 5.1.1) and available biochemical data for *P. monteilii*, the closest genetic relative of *P. alabamensis*, as identified by ANIb analysis

Species:	*P. alabamensis*		*P. plecoglossicida*	*P. fluorescens*	*P. monteilii*[1]
**Strain:**	**22-AL-CL-001ᵀ**	**USDA-ARS-USMARC-56711**	**Not specified***	**Not specified***	**CIP 104883ᵀ = DSM 14164ᵀ**
**Carbon Source:**					
Dextrin	-	-	-	-	NR
D-Maltose	-	-	-	-	-
D-Trehalose	-	-	-	+/-	-
D-Cellobiose	-	-	-	-	-
Gentiobiose	-	-	-	-	NR
Sucrose	-	-	-	-	-
D-Turanose	-	-	-	-	-
Stachyose	-	-	-	-	NR
D-Raffinose	-	-	-	-	-
α-D-Lactose	-	-	-	-	NR
D-Melibiose	-	-	-	-	-
β-Methyl-D-Glucoside	-	-	-	-	NR
D-Salicin	-	-	-	-	-
N-Acetyl-D-Glucosamine	-	-	-	+/-	-
N-Acetyl-β-D-Mannosamine	-	-	-	-	NR
N-Acetyl-D-Galactosamine	-	-	-	-	NR
N-Acetyl Neuraminic Acid	-	-	-	-	NR
α-D-Glucose	+	+	+	+/-	NR
D-Mannose	+	+/-	-	+/-	-
D-Fructose	+	+	-	+/-	+
D-Galactose	+	+/-	-	+	-
3-Methyl Glucose	-	-	-	-	NR
D-Fucose	+	+	-	+/-	-
L-Fucose	-	-	-	-	-
L-Rhamnose	-	-	-	-	-
Inosine	-	-	-	+/-	NR
D-Sorbitol	-	-	-	+/-	-
D-Mannitol	-	-	-	+/-	-
D-Arabitol	-	-	-	+/-	-
myo-Inositol	-	-	-	+	+

Supplementary Table 2 Continued

Species:	*P. alabamensis*		*P. plecoglossicida*	*P. fluorescens*	*P. monteilii*[1]
Strain:	22-AL-CL-001^T	USDA-ARS-USMARC-56711	Not specified*	Not specified*	CIP 104883^T = DSM 14164^T
**Carbon Source:**					
Glycerol	-	+	-	+/-	+
D-Glucose-6-PO4	-	-	-	-	NR
D-Fructose-6-PO4	+	+	-	-	NR
D-Aspartic Acid	-	-	-	+/-	NR
D-Serine	-	-	+/-	+/-	NR
Gelatin	-	-	-	-	-
Glycyl-L-Proline	-	-	-	-	NR
L-Alanine	-	-	+/-	+/-	+
L-Arginine	-	+	+/-	+/-	+
L-Aspartic Acid	-	+	+/-	+/-	+
L-Glutamic Acid	-	+	+/-	+	+
L-Histidine	-	-	-	+/-	+/-
L-Pyroglutamic Acid	-	-	+/-	+	NR
L-Serine	-	-	+/-	+/-	+
Pectin	-	-	-	-	NR
D-Galacturonic Acid	-	-	-	+/-	NR
L-Galactonic Acid Lactone	-	+/-	-	-	NR
D-Gluconic Acid	+	+	+/-	+/-	NR
D-Glucuronic Acid	-	-	-	-	NR
Glucuronamide	+	+	-	+/-	NR
Mucic Acid	+	+	+	+	NR
Quinic Acid	+	-	+/-	+	NR
D-Saccharic Acid	+	+	-	+	NR
p-Hydroxy-Phenylacetic Acid	-	-	-	+/-	NR
Methyl Pyruvate	-	+/-	+/-	-	NR
D-Lactic Acid Methyl Ester	-	-	-	-	NR
L-Lactic Acid	+	-	+/-	+	NR
Citric Acid	+	+	+	-	NR
α-Keto-Glutaric Acid	+	-	+/-	+	NR
D-Malic Acid	-	-	-	-	NR
L-Malic Acid	+	+	+	+	NR
Bromo-Succinic Acid	-	-	+/-	-	NR
Tween 40	-	-	-	-	NR

Supplementary Table 2 Continued

Species:	*P. alabamensis*		*P. plecoglossicida*	*P. fluorescens*	*P. monteilii*[1]
**Strain:**	**22-AL-CL-001^T**	**USDA-ARS-USMARC-56711**	**Not specified***	**Not specified***	**CIP 104883^T = DSM 14164^T**
**Carbon Source:**					
γ-Amino-Butyric Acid	+	+	+	+	NR
α-Hydroxy-Butyric Acid	-	-	-	-	NR
β-Hydroxy-D,L-Butyric Acid	-	-	+/-	+/-	NR
α-Keto-Butyric Acid	-	-	-	-	NR
Acetoacetic Acid	-	-	+/-	-	NR
Propionic Acid	-	-	+/-	+/-	NR
Acetic Acid	+	+	+	+	NR
Formic Acid	-	-	+/-	-	NR
pH 6	+	+	+	+	NR
pH 5	+	+	+/-	+/-	NR
1% NaCl	+	+	+	+	NR
4% NaCl	+	+	+/-	+/-	NR
8% NaCl	+	+	-	-	NR
1% Sodium Lactate	+	+	+	+	NR
Fusidic Acid	+	+	+/-	+/-	NR
D-Serine	+	+	-	+	NR

Supplementary Table 2 Continued

Species:	*P. alabamensis*		*P. plecoglossicida*	*P. fluorescens*	*P. monteilii*[1]
Strain:	22-AL-CL-001^{T}	USDA-ARS-USMARC-56711	Not specified*	Not specified*	CIP 104883^{T} = DSM 14164^{T}
**Chemical Sensitivity:**					
Troleandomycin	+	+	+/-	+/-	NR
Rifamycin SV	+	+	+	+	NR
Minocycline	-	+/-	-	-	NR
Lincomycin	+	+	+	+	NR
Guanadine HCl	+	+	+	+/-	NR
Niaproof 4	+	+	+	+	NR
Vancomycin	+	+	+	+	NR
Tetrazolium Violet	+	+	+	+	NR
Tetrazolium Blue	+	+	+	+	NR
Nalidixic Acid	+	+	+	+/-	NR
Lithium Chloride	+	+	+	+/-	NR
Potassium Tellurite	+	+	+	+	NR
Aztreonam	+	+	+/-	+/-	NR
Sodium Butyrate	-	-	+/-	-	NR
Sodium Bromate	+	+	+/-	+/-	NR

[1]Biochemical profiling data for *P. monteilii* was obtained from Elomari et al., 1997.

Positive reactions are designated (+); negative reactions are designated (-); indeterminate reactions are designated (+/-); reactions which were not reported in Elomari et al., 1997 are designated (NR).

*Species reference records within the Biolog MicroLog™ M System database (v. 5.1.1) do not contain strain information.
